# Functional monovalency amplifies the pathogenicity of anti-MuSK IgG4 in myasthenia gravis

**DOI:** 10.1073/pnas.2020635118

**Published:** 2021-03-22

**Authors:** Dana L. E. Vergoossen, Jaap J. Plomp, Christoph Gstöttner, Yvonne E. Fillié-Grijpma, Roy Augustinus, Robyn Verpalen, Manfred Wuhrer, Paul W. H. I. Parren, Elena Dominguez-Vega, Silvère M. van der Maarel, Jan J. Verschuuren, Maartje G. Huijbers

**Affiliations:** ^a^Department of Human Genetics, Leiden University Medical Center, 2333 ZA, Leiden, The Netherlands;; ^b^Department of Neurology, Leiden University Medical Center, 2333 ZA, Leiden, The Netherlands;; ^c^Center of Proteomics and Metabolomics, Leiden University Medical Center, 2333 ZA, Leiden, The Netherlands;; ^d^Department of Immunology, Leiden University Medical Center, 2333 ZA, Leiden, The Netherlands;; ^e^Lava Therapeutics, 3584 CM, Utrecht, The Netherlands

**Keywords:** IgG4, autoimmunity, myasthenia gravis, MuSK, monoclonal antibodies

## Abstract

Human immunoglobulin (Ig) G4 usually displays antiinflammatory activity, and observations of IgG4 autoantibodies causing severe autoimmune disorders are therefore poorly understood. In blood, IgG4 naturally engages in a stochastic process termed “Fab-arm exchange” in which unrelated IgG4s exchange half-molecules continuously. The resulting IgG4 antibodies are composed of two different binding sites, thereby acquiring monovalent binding and inability to cross-link for each antigen recognized. Here, we demonstrate that this process amplifies autoantibody pathogenicity in a classic IgG4-mediated autoimmune disease: muscle-specific kinase (MuSK) myasthenia gravis. In mice, monovalent anti-MuSK IgG4s caused rapid and severe myasthenic muscle weakness, whereas the same antibodies in their parental bivalent form were less potent or did not induce a phenotype. Mechanistically this could be explained by opposing effects on MuSK signaling. Isotype switching to IgG4 in an autoimmune response thereby may be a critical step in the development of disease. Our study establishes functional monovalency as a pathogenic mechanism in IgG4-mediated autoimmune disease and potentially other disorders.

## Significance

An expanding group of autoimmune diseases is now recognized to be hallmarked by pathogenic IgG4 autoantibodies. IgG4 has the unique ability to exchange Fab-arms, rendering it bispecific and functionally monovalent. Here we show that autoantibody functional monovalency significantly amplifies the pathogenicity of IgG4 autoantibodies using patient-derived monoclonal antibodies in an in vivo model of MuSK myasthenia gravis. Therefore, subclass switching to predominant IgG4 autoantibodies is a critical step in the development of MuSK myasthenia gravis. This new mechanism in autoimmunity is also potentially relevant to 29 other IgG4-mediated autoimmune diseases known to date, allergy and other disease settings where IgG4 antibodies contribute to pathology.

Recently, a growing class of antibody-mediated autoimmune diseases characterized by predominant pathogenic immunoglobulin (Ig) G4 responses has been described (1–4). IgG4 is a peculiar antibody with unique characteristics. It is, for example, unable to activate complement and has a low affinity for Fcγ receptors on immune cells (5, 6). It is therefore considered antiinflammatory, and the pathogenicity of IgG4 autoantibodies is sometimes questioned. IgG4 molecules furthermore have the unique ability to stochastically exchange half-molecules with other IgG4s in a dynamic process called “Fab-arm exchange” (7). This is an efficient process resulting in the vast majority of IgG4 molecules in circulation being bispecific and functionally monovalent for each antigen recognized. Whether the unique functional characteristics of IgG4 (like Fab-arm exchange) influence their pathogenicity in IgG4 autoimmune diseases is not known.

Myasthenia gravis (MG) with antibodies against muscle-specific kinase (MuSK) is one of the first recognized IgG4-mediated autoimmune diseases. MuSK autoantibodies are predominantly of the IgG4 subclass, although anti-MuSK IgG1 and IgG3 may be present concurrently at lower titers (8). Anti-MuSK IgG4s induce MG in a dose-dependent manner both in patients and in mice (9, 10). MuSK, a receptor tyrosine kinase, has a crucial role in establishing and maintaining neuromuscular junctions (NMJs) by orchestrating postsynaptic acetylcholine receptor (AChR) clustering, which is critical for neurotransmission (11). Most MuSK autoantibodies bind the extracellular N-terminal Ig-like 1 domain and thereby block the activation of MuSK by low-density lipoprotein receptor-related protein 4 (Lrp4) and agrin (8, 12–16). This eventually leads to disassembly of densely packed AChRs in the NMJ, failure of neurotransmission, and, consequently, muscle weakness (10, 17–20). In vitro characterization of monoclonal antibodies derived from MuSK MG patients furthermore suggests that the valency of MuSK antibodies determines their effects on MuSK signaling (21–23). Monovalent Fab fragments recapitulate the inhibitory effects of patient-purified IgG4 on MuSK signaling in vitro (12, 13, 21, 23). Surprisingly, monospecific bivalent MuSK antibodies acted oppositely, as (partial) agonists (21). To investigate whether IgG4 predominance is critical for disease development in IgG4-mediated autoimmunity and study the role of Fab-arm exchange and autoantibody valency, we generated stable bispecific functionally monovalent MuSK antibodies and their monospecific bivalent equivalents and assessed their pathogenicity in NOD/SCID mice.

## Results

### Generation of Patient-Derived Stable Bispecific Monovalent IgG4 MuSK Antibodies.

**Fig. 1.**
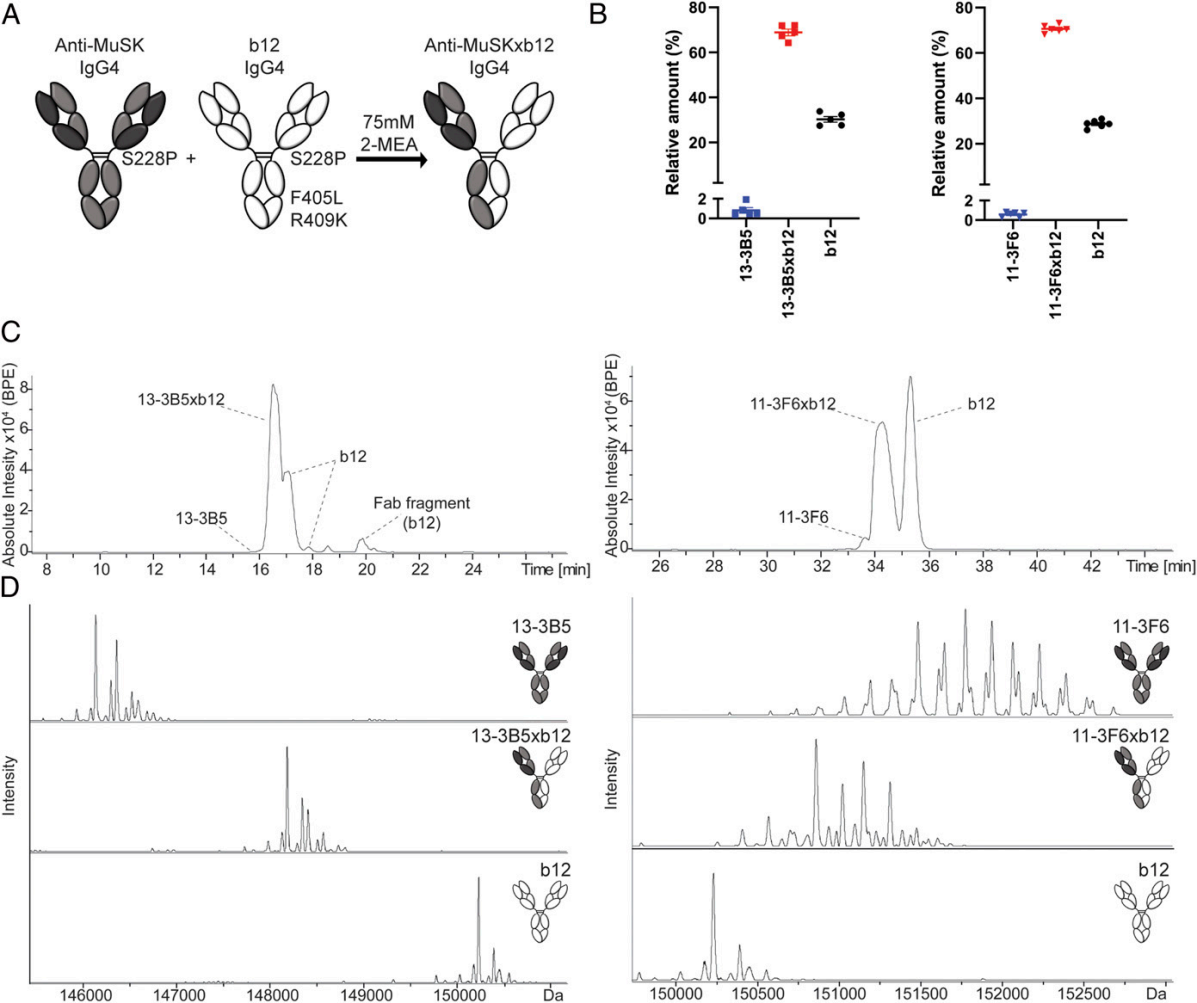
Generation of stable, MuSK MG patient-derived, bispecific, monovalent recombinant IgG4 MuSK antibodies. (*A*) Graphic depiction of the amino acid substitutions introduced for controlled Fab-arm exchange with IgG4. To make all antibodies resistant to Fab-arm exchange in vivo, serine 228 was converted to a proline, strengthening the interaction in the hinge region. To stabilize bispecific (monovalent) IgG4s, a leucine (L) at position 405 and a lysine (K) at position 409 were introduced in the b12 antibody to create complementary amino acids with the phenylalanine (F) and arginine (R) in the anti-MuSK IgG4s, respectively. The interaction between the heterodimers is stronger than that between the homodimers, preventing in vivo and further in vitro exchange of the bispecific IgG4. (*B*) The exchange reaction with 75 mM 2-mercaptoethylamine (2-MEA) yielded high exchange efficiency with <1% contamination of the original monospecific (bivalent) anti-MuSK IgG4 for both clones (*n* = 5). The b12 exchange partner was added in excess, yielding a remnant of ∼30% after exchange. Representative examples of (*C*) base peak electropherogram (BPE) and (*D*) deconvoluted mass spectra of 11–3F6 and 13–3B5, respectively. The materials for 11–3F6xb12 exchange efficiency tests came from productions in both CHO and HEK293 cells. Representative images of 11–3F6 and 11–3F6xb12 deconvoluted mass spectra are from a production in CHO cells. Data represent mean ± SEM.

To investigate the role of MuSK antibody valency on their pathogenicity in vivo, pure and stable bispecific MuSK antibodies are needed. Remaining monospecific MuSK antibodies may have confounding effects, e.g., by competing for binding with MuSK and thereby masking effects of the bispecific MuSK antibodies. We therefore adapted the controlled Fab-arm exchange (cFAE) method to IgG4 (24, 25). In addition to two previously identified patient-derived recombinant MuSK antibodies (13-3B5 and 11–3F6) (21), the b12 antibody was chosen to generate an innocuous arm in the bispecific MuSK antibody because its antigen (the HIV-1 envelope protein gp120) does not exist in the model systems used (26). To stabilize the antibodies under physiological conditions, the serine (S) at amino acid position 228 was converted into a proline (P) in the IgG4 heavy chain of the anti-MuSK antibodies (Fig. 1*A*). The b12 antibody was made suitable for efficient cFAE by altering S228P, F405L, and R409K in its IgG4 heavy chain. For clarity, the parental monospecific IgG4 will be designated as bivalent and the bispecific IgG4 as monovalent toward MuSK for the remainder of this paper.

The exchange efficiency and residual amount of the bivalent (monospecific) anti-MuSK antibodies were determined with capillary electrophoresis (CE) hyphenated with mass spectrometry (MS) (27). The monovalent (bispecific) IgG4 was separated from its two bivalent parents with CE, permitting reliable determination of their relative amounts down to 0.5% (Fig. 1*C* and *SI Appendix*, Fig. S1). The purity of the separated fractions was confirmed with MS (Fig. 1*D*). The efficiency of exchange was high for both antibodies, with <1% of bivalent MuSK antibody remaining. The b12 antibody was added in molar excess to drive exchange of the MuSK antibody to completion, and on average ∼30% excess b12 IgG4 is therefore apparent in the CE and MS analyses (Fig. 1*B*).

### Monovalent MuSK Antibodies Are More Pathogenic than Bivalent MuSK Antibodies in Mice.

**Fig. 2.**
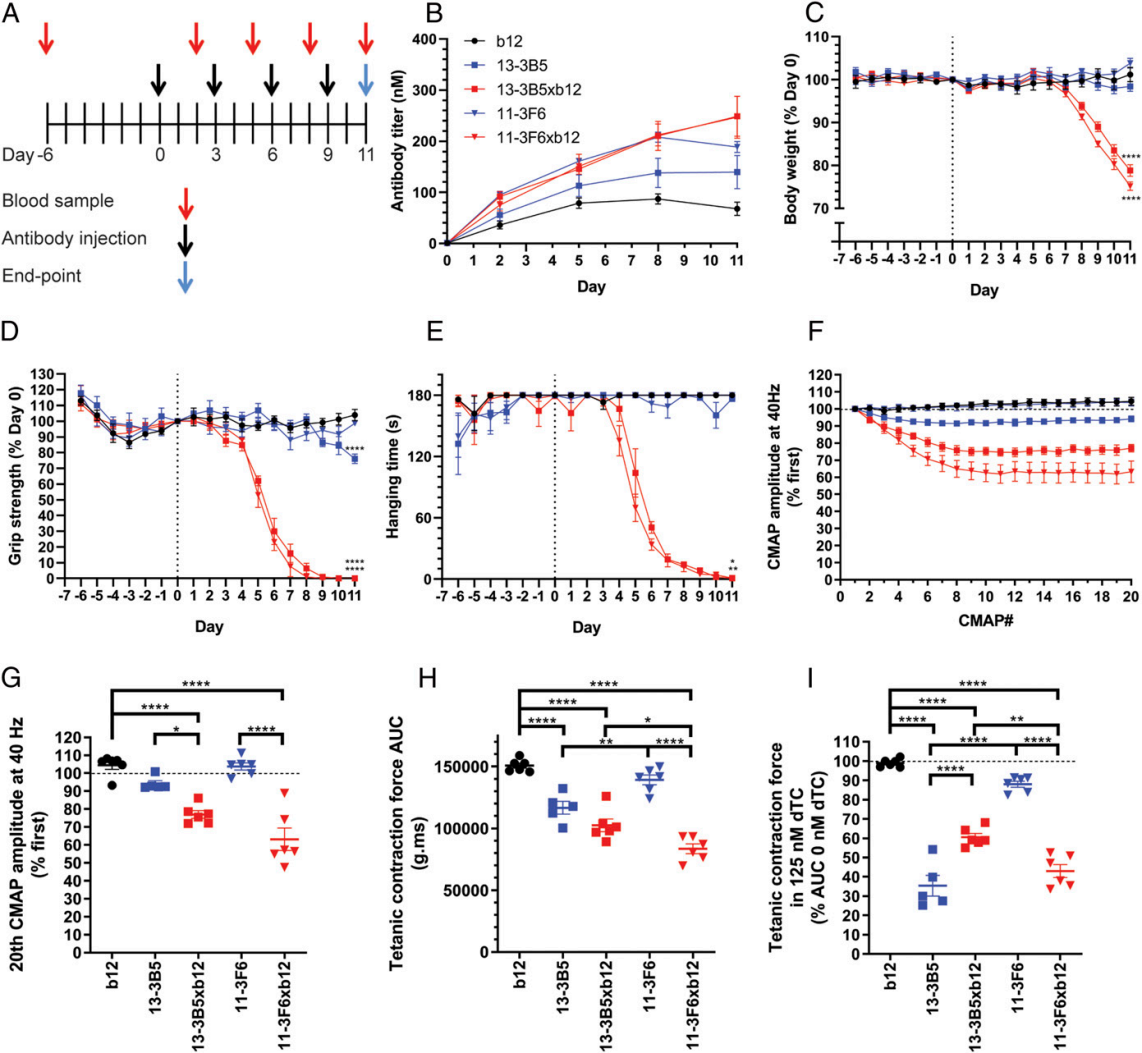
Monovalent MuSK antibodies induce rapid onset, progressive myasthenic symptoms in mice, whereas bivalent MuSK antibodies do not. (*A*) Experimental design of passive transfer with 2.5 mg/kg recombinant antibody. (*B*) Serum antibody titers confirmed exposure. (*C*–*E*) Monovalent, but not bivalent, MuSK antibodies induced progressive weight loss and muscle weakness. Bivalent 13–3B5 induced a delayed, mild loss of grip strength. (*F* and *G*) Monovalent, but not bivalent, MuSK antibodies induced a significantly larger CMAP decrement upon repetitive stimulation at 40 Hz compared with the b12 control. (*H*) Ex vivo tetanic contraction force of the diaphragm was reduced by 13–3B5, 13–3B5xb12, and 11–3F6xb12 compared with b12. (*I*) The safety factor of neuromuscular transmission was assessed in the presence of 125 nM dTC. Exposure to all MuSK antibodies reduced the safety factor compared with the b12 control, although for 11–3F6 this was only a trend (*P* = 0.079). 11–3F6 and 11–3F6xb12: *n* = 6; 13–3B5: *n* = 5; 13–3B5xb12 and b12: *n* = 6 (hanging time *n* = 5). Data represent mean ± SEM. One-way ANOVA with Šidák-corrected comparisons for all parameters (*C*, *D*, *G*, *H*, and *I*) except hanging time, for which Kruskal–Wallis test was used (*C*). *P < 0.05, **P < 0.01, *** <0.001, ****P < 0.0001 compared with b12 group, unless otherwise specified.

Passive transfer of MuSK antibodies to NOD/SCID mice is a well-established method to investigate the development of myasthenic muscle weakness without confounding immune reactions to human antibodies (10, 28). Immunostaining of whole-mount levator auris longus muscle confirmed that both mono- and bivalent MuSK antibodies bound MuSK at the NMJ, while the control b12 antibody did not (*SI Appendix*, Fig. S2*A*). The in vivo half-life ranged between 38 and 63 h and varied by antibody (*SI Appendix*, Fig. S3 *A* and *B*). To ensure continuous in vivo exposure, an injection regimen of every 3 to 4 d was chosen for the passive transfer experiments. The minimum dose required to induce progressive phenotypical myasthenic symptoms was determined with monovalent 11–3F6xb12 IgG4. A dose of 2.5 mg/kg every 3 to 4 d resulted in progressive muscle weakness starting after about 5 d, leading to 20% body weight loss after 11 d (*SI Appendix*, Fig. S3 *D*–*F*). No clinical myasthenic muscle weakness or weight loss was observed at the 1.25 mg/kg dose. The serum antibody levels in these latter mice were approximately 10-fold lower compared with the 2.5 mg/kg regimen, indicating at these lower doses the pharmacokinetics do not change linearly (*SI Appendix*, Fig. S3*C*). To compare the pathogenicity of monovalent vs. bivalent MuSK antibodies, we injected the bivalent or monovalent MuSK antibodies or the b12 control at 2.5 mg/kg every 3 d (Fig. 2*A*).

Both monovalent anti-MuSK IgG4s induced rapid and severe myasthenic muscle weakness and body weight loss (Fig. 2 *C*–*E* and *SI Appendix*, Fig. S4 *B*–*D* for individual traces). The mice lost nearly all their grip strength and showed progressive fatigable muscle weakness in the inverted mesh test, starting within 1 wk (Fig. 2 *D* and *E*). After a week, the mice also started to rapidly lose weight, likely as a consequence of the previously described bulbar muscle weakness, which makes it difficult to chew and swallow food (Fig. 2*C*) (18, 29, 30). In sharp contrast, the bivalent 11–3F6 and 13–3B5 did not induce weight loss. Bivalent 11–3F6 furthermore did not induce any sign of clinical muscle weakness in grip strength or inverted mesh hanging time (Fig. 2 *D* and *E*). The bivalent 13–3B5 induced a mild, but statistically significant, loss of grip strength only on the final day of the experiment (Fig. 2*D*). Furthermore, three of five mice in this group could not complete the 3 min hanging test on day 10 and/or 11, while all mice in the control group could (Fig. 2*E* and *SI Appendix*, Fig. S4*D*).

To assess muscle function in more detail, repetitive nerve stimulation electromyography (EMG) on the left calf muscles was performed on the final day of the experiment. Decrement of the compound muscle action potential (CMAP) is a clinical electrophysiological hallmark of MG and is caused by loss of transmission in a progressive number of NMJs during the stimulations (31). Both monovalent MuSK antibodies induced 20 to 30% decrement at 40 Hz simulation [approximately the physiological firing rate of motor neurons (32)], indicating severe muscle fatigability (Fig. 2 *F* and *G*). This decrement was significantly larger compared with the bivalent parent of both IgG4s. Notably, the bivalent antibodies did not differ from the b12 control group, indicating normal muscle functioning.

To monitor in vivo exposure to the antibodies, serum samples were taken before the first injection and 2 d after every following injection. Serum titers varied between individual mice (Fig. 2*B* and *SI Appendix*, Fig. S4*A*), indicating individual differences in pharmacokinetics. Specifically, serum levels of the b12 antibody were lower compared with the different anti-MuSK clones, despite identical dosing. The levels of the MuSK antibodies were quite comparable, except at the final stage of the experiment, where titers of the bivalent IgG4s plateaued, while the monovalent anti-MuSK IgG4 serum levels continued to rise. Possible explanations could be the body weight and volume loss which mice injected with monovalent MuSK antibodies experienced at this stage or the kinetic differences between monovalent and bivalent MuSK antibodies due to receptor-mediated internalization and degradation.

To confirm that the IgG4s were stable in vivo, serum was collected at the final day of the experiment and analyzed with CE-MS. All five antibodies were successfully retrieved from the circulation and found to be intact at the end of the experiment (*SI Appendix*, Fig. S5). The only modification observed was the in vivo removal of C-terminal lysine for 11–3F6 and 11–3F6xb12 (*SI Appendix*, Fig. S5 *C* and *E*). C-terminal lysine clipping of IgGs is a well-known natural process, which does not affect antigen-binding ability (33). For both monovalent MuSK antibodies, the proportion of excess b12 antibody seems to be reduced after injection, as seen in the base peak electropherogram (BPE) (*SI Appendix*, Fig. S5 *D* and *E*). The parental b12 control antibody therefore seems to be cleared slightly faster, but this is unlikely to have had an effect on the model as such. No other species or degradation products could be detected, indicating that the antibodies remained stable in vivo and ruling out confounding effects, such as disassembly of bivalent MuSK antibodies.

In conclusion, all mice exposed to monovalent MuSK IgG4 rapidly developed severe myasthenic symptoms on all in vivo outcome measures. Mice exposed to the same dosing of bivalent MuSK IgG4 did not show overt phenotypical myasthenia. Thus, the functional monovalency of these bispecific (Fab-arm exchanged) anti-MuSK IgG4s makes them much more pathogenic than their monospecific equivalents with functional bivalency for MuSK.

### Monovalent MuSK Antibodies Induce Reduced Ex Vivo Contraction Force Compared with Bivalent MuSK Antibodies.

To further characterize muscle function, the contraction force upon repetitive nerve stimulation was examined on the left hemidiaphragm ex vivo. Of note, functional effects on the diaphragm may be larger compared with other muscles because it is directly exposed to the antibodies upon intraperitoneal (i.p.) injection. Stimulation of the phrenic nerve at 40 Hz for 7 s resulted in tetanic contraction. Quantification of the area under the curve revealed similar patterns between groups as seen with the CMAP decrement in the calf muscles. The monovalent MuSK antibodies reduced the contraction force most severely (Fig. 2*H*). Baseline contraction force upon exposure to bivalent 11–3F6 was indistinguishable from the b12 control group, while mice that received bivalent 13–3B5 showed a mild but statistically significant reduction of contraction force. Furthermore, the diaphragm contraction force was lower for the monovalent compared with their bivalent counterparts, although this was statistically significant only for 11–3F6xb12.

Subclinical signs of myasthenic muscle pathophysiology can be investigated by assessing the safety factor of neuromuscular transmission with the reversible AChR blocker d-tubocurarine (dTC). In the b12 control group, the tetanic contraction force of the diaphragm was not reduced by 125 nM dTC, indicating a healthy safety factor (Fig. 2*I*). Both monovalent anti-MuSK IgG4s substantially reduced the safety factor. In contrast, bivalent 11–3F6 did not show a statistically significant reduction of tetanic contraction, although a trend was seen (*P* = 0.079). Diaphragms of mice exposed to bivalent 13–3B5 were affected by 125 nM dTC, indicating that although the clinical phenotype caused by 13–3B5 is mild, the NMJs already have substantial loss of AChR and are in a state of subclinical myasthenia (Fig. 2*I*). Taken together, monovalent MuSK IgG4 antibodies caused significant reduction in contraction force and safety factor of neurotransmission. Bivalent MuSK antibodies induced subclinical signs of myasthenia, but the extent was antibody dependent.

### Monovalent and Bivalent MuSK Antibodies Affect Neuromuscular Junction Morphology.

**Fig. 3.**
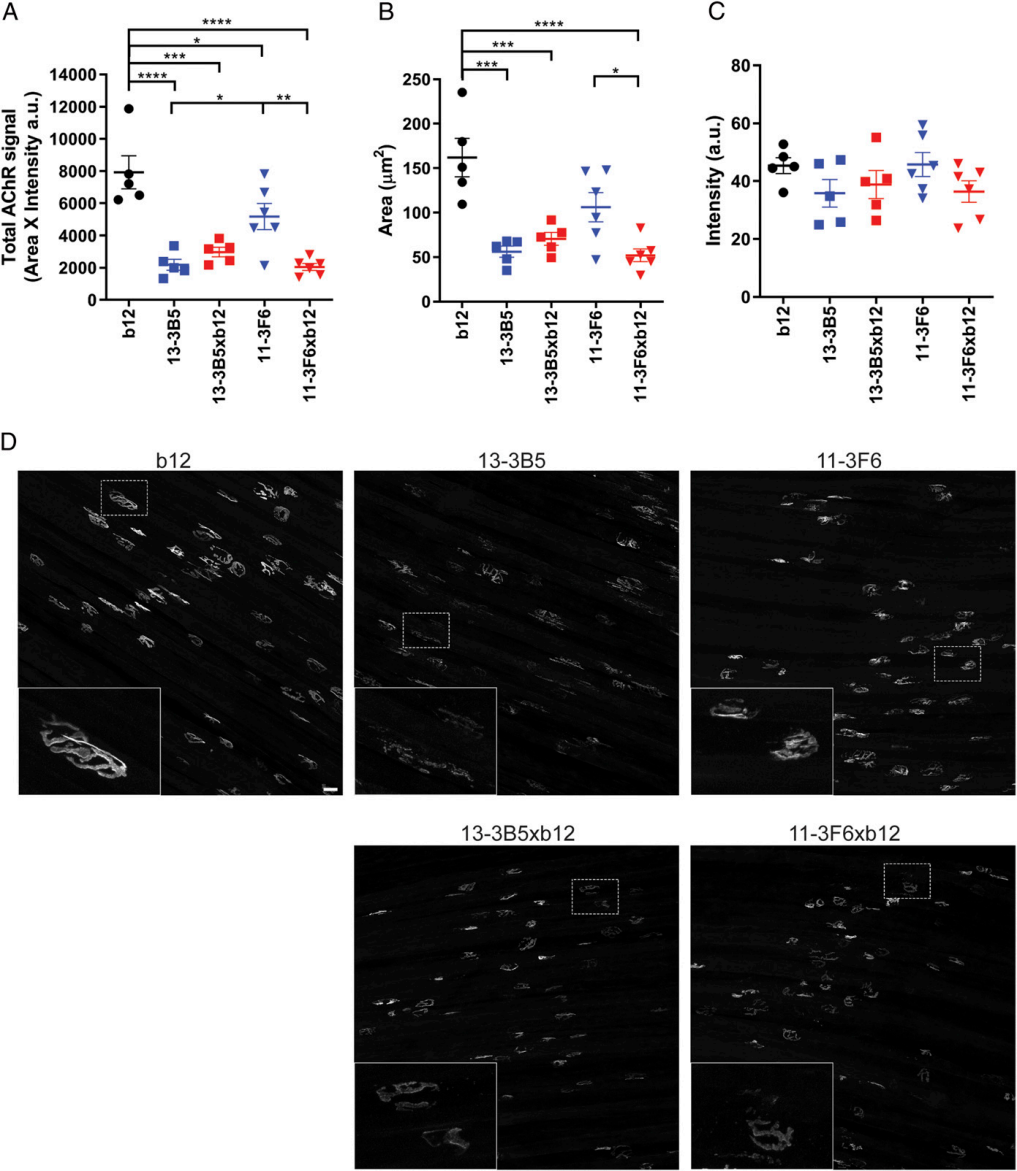
Monovalent and bivalent MuSK antibodies impair NMJ morphology to a different extent. To visualize the postsynaptic NMJ, AChRs were stained with AF488-BTX on diaphragm muscle preparations. Twenty randomly selected NMJs per diaphragm were analyzed and averaged. (*A*) Total AChR signal was calculated by multiplying the positive area with the average intensity per NMJ. Both bivalent and monovalent MuSK antibodies significantly reduced the total AChR signal compared with the b12-treated mice. Bivalent 13–3B5 and monovalent 11–3F6xb12 reduced the total AChR signal to a greater extent compared with monospecific 11–3F6. Bivalent 13–3B5 did not significantly differ from monovalent 13–3B5xb12. (*B*) To assess the size of the NMJs, a threshold was applied, and the area of the positive signal was quantified. Exposure to monovalent MuSK antibodies or bivalent 13–3B5 resulted in less signal reaching the threshold and therefore smaller NMJs. Monovalent 11–3F6xb12 also caused significantly smaller NMJs compared with bivalent 11–3F6. (*C*) The average intensity of AChR staining surpassing the threshold did not significantly differ between groups. (*D*) Representative maximum projections per condition with insets. (Scale bar, 25 µm.) 11–3F6 and 11–3F6xb12 *n* = 6, 13–3B5, 13–3B5xb12 and b12 *n* = 5. Data represent mean ± SEM. One-way ANOVA with Šidák-corrected comparisons for all parameters: *P <0.05, **P <0.01, ***P <0.001, ****P <0.0001.

A fragmented and reduced AChR area at NMJs is a well-described pathological feature, causing muscle weakness in MuSK MG animal models (10, 17, 18, 20, 34–37). To investigate the postsynaptic NMJ morphology after in vivo exposure to mono- and bivalent MuSK antibodies, AChRs were stained in whole-mount preparations of the right hemidiaphragm and epitrochleoanconeus (ETA) (Fig. 3 and *SI Appendix*, Fig. S6 *A* and *D*). Quantification of the total AChR signal per NMJ revealed that both monovalent anti-MuSK IgG4s and the bivalent 13–3B5 caused a strong reduction of AChRs, while mice exposed to bivalent 11–3F6 had more remaining AChRs compared with monovalent 11–3F6xb12 and bivalent 13–3B5 (Fig. 3*A*). The area of positive AChR signal was also significantly reduced in the muscles from mice treated with monovalent anti-MuSK IgG4s or bivalent 13–3B5 (Fig. 3*B*). The average intensity of postthreshold AChR staining was not statistically different between the conditions, likely due to animal-to-animal variance in these small groups (Fig. 3*C*). In sum, exposure to mono- and bivalent MuSK antibodies reduced the number of postsynaptic AChRs, with the effect of the bivalent MuSK antibodies being antibody dependent.

Loss of appropriate pre- and postsynaptic alignment is also known to contribute to the NMJ dysfunction seen in MG animal models (2, 10, 17). Therefore, this was assessed in the ETA muscle of a subset of animals (*SI Appendix*, Fig. S6). The intensity and morphology of the presynaptic SV2 signal were not affected by exposure to these mono- and bivalent anti-MuSK IgG4s (*SI Appendix*, Fig. S6*B*). Denervation assessed by the colocalization of the presynaptic SV2 signal with the postsynaptic AChR signal revealed a similar pattern as seen with the postsynaptic AChR signal (*SI Appendix*, Fig. S6 *A* and *C*). Overall, these data suggest that postsynaptic pathology of the NMJ is the main cause of the neuromuscular dysfunction induced by these MuSK antibodies and that loss of AChR area results in less alignment with the presynapse.

### Antibody-Dependent Pathogenicity of Parental Bivalent MuSK Antibodies.

**Fig. 4.**
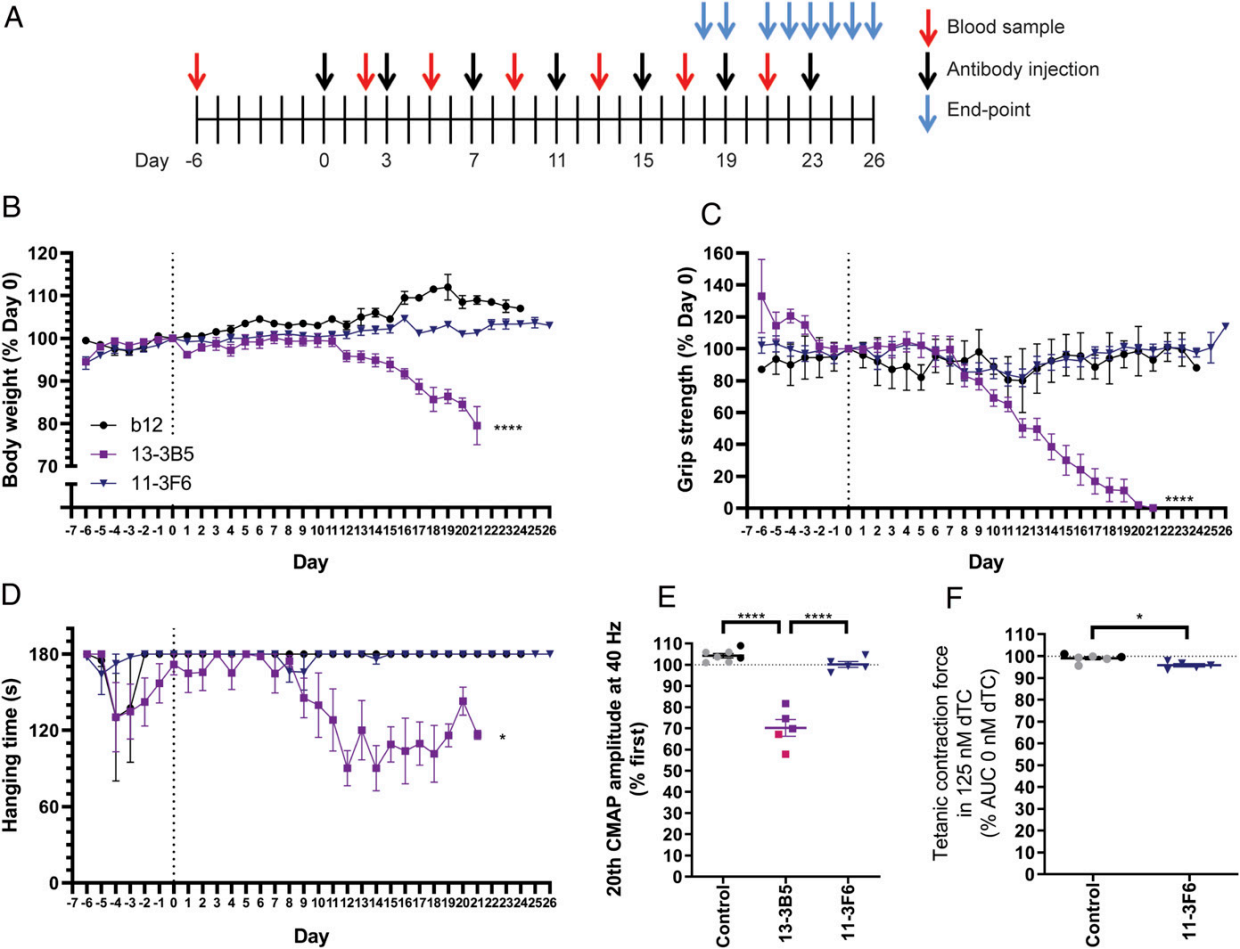
Antibody-dependent pathogenicity of bivalent MuSK antibodies. (*A*) Experimental design of passive transfer in NOD/SCID mice. Mice exposed to bivalent 13–3B5 started to (progressively) lose weight (*B*), grip strength (*C*), and hanging time on the inverted mesh (*D*) in the third and second week of the experiment, respectively. Mice exposed to bivalent 11–3F6 did not show progressive loss on these parameters (*B*–*D*). (*E*) Mice exposed to bivalent 13–3B5 showed a significant ∼30% CMAP decrement at the end point, while bivalent 11–3F6 did not induce a decrement. (*F*) Contraction force of the diaphragm was significantly, but very mildly (∼3% reduction), affected by 125 nM dTC for mice exposed to bivalent 11–3F6. Data represent mean ± SEM 13–3B5: 5 mg/kg (pink): *n* = 2; 10 mg/kg (purple): *n* = 4 (except for hanging time on inverted mesh and EMG: *n* = 3); 11–3F6: 10 mg/kg: *n* = 5; b12 (black): *n* = 2, combined with untreated (gray): *n* = 5 for CMAP or *n* = 4 for tetanic contraction).

To investigate whether both bivalent MuSK antibodies could be pathogenic, albeit at a higher dose or with prolonged exposure, we increased the dosing to 5 mg/kg and 10 mg/kg and extended the duration of the passive transfer to 3 wk (Fig. 4*A*). The mice exposed to bivalent 13–3B5 displayed overt signs of muscle weakness on grip strength and inverted mesh from days 9 to 12 onwards and progressively lost weight in week 3 (Fig. 4 *B*–*D* and *SI Appendix*, Fig. S7 *B*–*D* for individual traces). Grip strength slowly declined over a period of a week to a near complete loss on the final days of the experiment (Fig. 4*C*). Hanging time on the inverted mesh did not show a clear progressive decline in the majority of mice exposed to bivalent 13–3B5 (*SI Appendix*, Fig. S7*D*). Finally, exposure to bivalent 13–3B5 resulted in a significant CMAP decrement compared with both the control group and bivalent 11–3F6 (Fig. 4*E*). In sum, prolonged exposure to bivalent 13–3B5 IgG4 can cause progressive and severe myasthenic muscle weakness. Interestingly, the onset of symptoms is later and progresses more slowly compared with monovalent 13–3B5xb12.

Mice exposed to bivalent 11–3F6 did not show signs of myasthenic muscle weakness on any of the in vivo parameters at higher dose and after prolonged exposure (Fig. 4 *B*–*E*). To detect possible subclinical pathology, the safety factor of neurotransmission was assessed in the diaphragm. For reference, the relative tetanic contraction force of animals treated with the b12 antibody was supplemented with untreated healthy animals. The safety factor of mice exposed to 10 mg/kg of bivalent 11–3F6 was slightly (∼3%), but significantly reduced compared with control muscle (Fig. 4*F*). The effect size of the reduced safety factor seen in mice exposed to 2.5 mg/kg of bivalent 11–3F6 for 11 d therefore does not seem to worsen over time and with higher dose (Fig. 2*I*). Antibody titers were comparable between mice injected with bivalent 13–3B5 or 11–3F6 (*SI Appendix*, Fig. S7*A*). The differential effects between 13–3B5 and 11–3F6 therefore confirm that bivalent MuSK antibodies can be pathogenic, but their pathogenic potential is antibody dependent.

### Monovalent MuSK Antibodies Inhibit MuSK Signaling In Vitro.

**Fig. 5.**
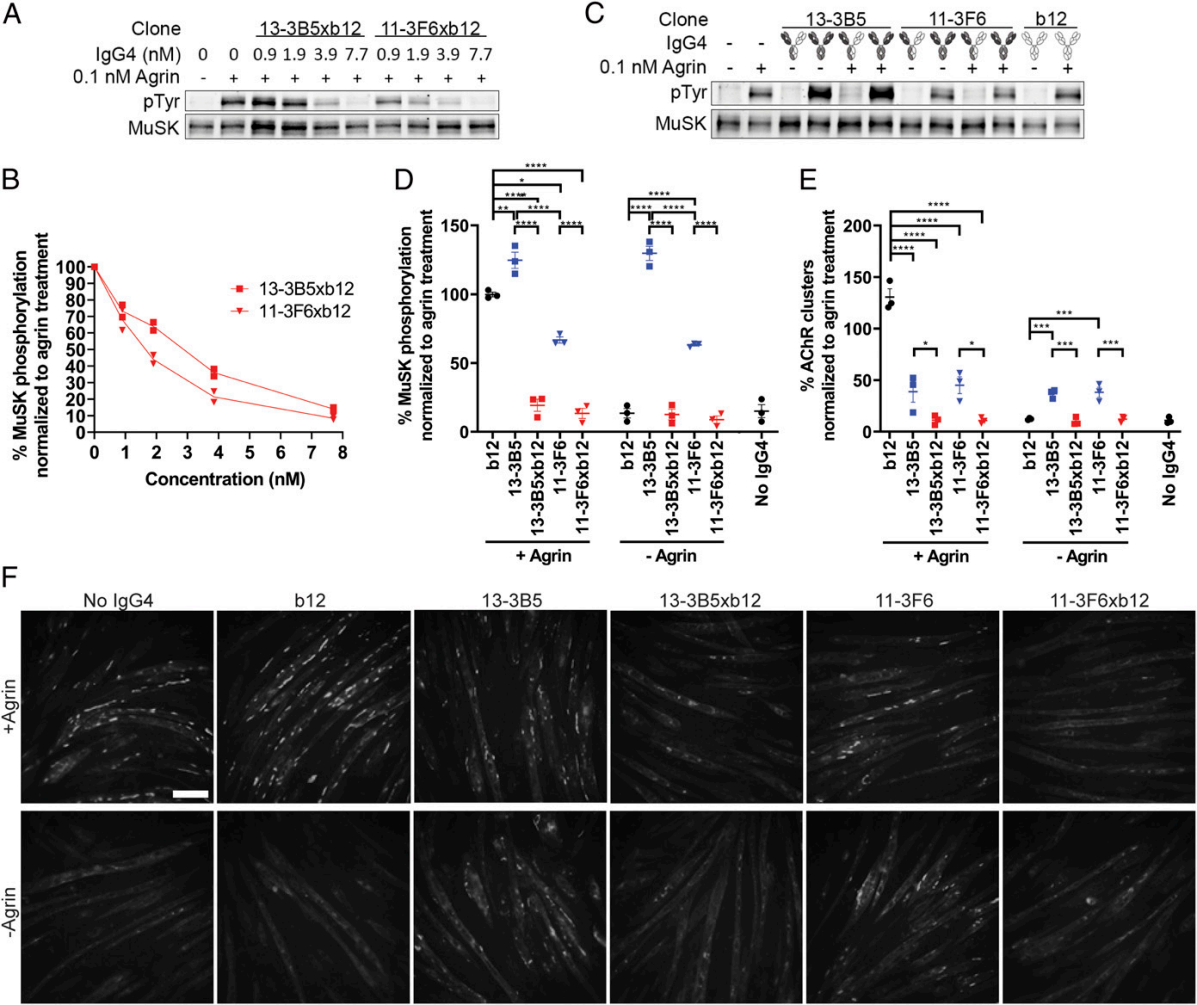
Monovalent IgG4 MuSK antibodies abolish agrin-induced signaling of the agrin-Lrp4-MuSK cascade. (*A* and *B*) Monovalent MuSK antibodies inhibited agrin-induced MuSK phosphorylation in a concentration-dependent manner in C2C12 myotubes (*n* = 2). Maximum inhibition was reached at 7.7 nM. (*C* and *D*) Bivalent MuSK antibodies activated MuSK phosphorylation independent of agrin, while monovalent MuSK antibodies fully inhibited agrin-induced MuSK phosphorylation. The b12 control did not affect MuSK phosphorylation. To correct for loading differences, the phosphotyrosine (pTyr) signal was divided by the amount of MuSK that was immunoprecipitated and normalized to the agrin-only condition per replicate (*n* = 3). (*E* and *F*) Monovalent MuSK antibodies completely inhibited agrin-induced AChR clustering (visualized by AF488-BTX staining). Bivalent MuSK antibodies partially induced AChR clustering independent of agrin, but partially inhibited agrin-induced clustering. Large (>15 µm^2^) AChR clusters were counted and normalized to the agrin-only condition per replicate (*n* = 3). Data represent mean ± SEM. One-way ANOVA with Šidák-corrected comparisons. *P <0.05, **P <0.01, ***P <0.001, ****P <0.0001. (Scale bar, 100 µm.)

C2C12 myotubes contain all the muscle-specific machinery to interrogate the agrin-Lrp4-MuSK-signaling cascade in vitro, except for neural agrin. Addition of agrin to these cultures activates this cascade, leading to MuSK phosphorylation and AChR clustering (38–40). Monovalent MuSK antibodies were found to inhibit agrin-induced MuSK phosphorylation in a concentration-dependent manner (Fig. 5 *A* and *B*). Bivalent anti-MuSK IgG4 in stark contrast (partially) induced MuSK phosphorylation and AChR clustering in both the absence and the presence of agrin (Fig. 5 *C*–*F*). These opposing effects extend previous observations using monovalent Fab fragments (21, 23). The bivalent 13–3B5 and 11–3F6 antibodies induced MuSK phosphorylation to different extents, with 13–3B5 reaching supra-agrin levels, while maximum phosphorylation induced by 11–3F6 reached about 70% of the level of agrin. Bivalent anti-MuSK antibodies induced similar amounts of large AChR clusters (>15 µm^2^), which are considered most mature and therefore relevant (41). However, 13–3B5 also induced more smaller clusters compared with 11–3F6 (*SI Appendix*, Fig. S2*B*). In summary, monovalent MuSK IgG4 abolished agrin-Lrp4-MuSK signaling in vitro, whereas bivalent anti-MuSK IgG4 partially activated this signaling, independent of agrin.

## Discussion

**Fig. 6.**
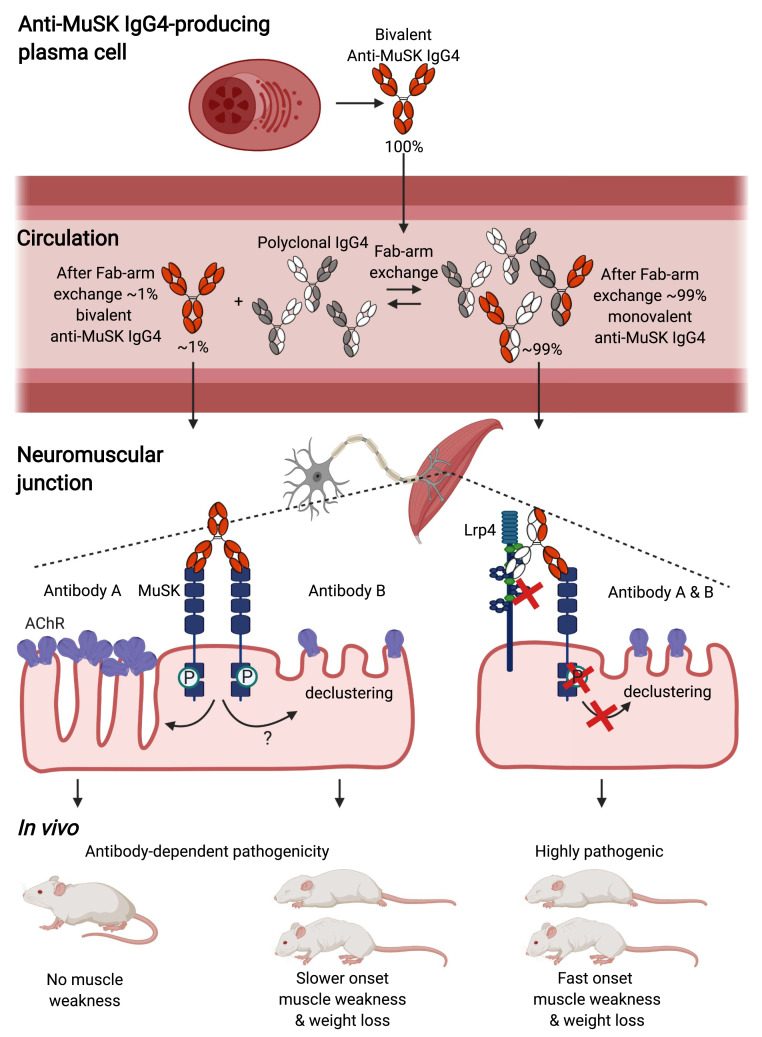
Functional monovalency amplifies the pathogenicity of anti-MuSK IgG4. Anti-MuSK IgG4 is produced as monospecific bivalent antibody. Upon reaching the circulation, Fab-arm exchange renders ∼99% of IgG4 MuSK antibodies bispecific and functionally monovalent. Monovalent MuSK antibodies reach NMJs and, upon binding to MuSK, block its function and induce AChR declustering, resulting in fast-onset progressive myasthenia. Monospecific functionally bivalent MuSK antibodies dimerize and activate MuSK signaling independent of agrin. Depending on the clone, this direct effect on MuSK either results in more slowly progressing myasthenia due to depletion of AChRs, by a yet-to-be-discovered mechanism, or induces no clinical phenotype with largely intact NMJs. *P*: phosphorylation. This figure was created with BioRender.com.

In this study, we demonstrate a new disease mechanism in autoimmunity related to the unique feature of IgG4 to undergo Fab-arm exchange. We provide evidence that autoantibody functional monovalency for MuSK amplifies the in vivo pathogenicity of IgG4 MuSK antibodies. Mechanistically, this may be explained by the respective antagonistic vs. agonistic effects of the Fab-arm exchanged monovalent and parental bivalent IgG4 antibodies on the MuSK-signaling cascade. The sequence of events driving IgG4 autoantibody pathogenicity and the main conclusions of this study are summarized in Fig. 6. Thus, class switching to IgG4 not only may be a characteristic of IgG4-mediated autoimmunity, but also a crucial step in symptom manifestation.

IgG4 is the only human antibody subclass able to undergo Fab-arm exchange under physiological conditions (42). It furthermore has limited ability to stimulate inflammation. The relevance of (Fab-arm exchanged) IgG4 is thought to be inhibition of ongoing detrimental immune responses against exogenous antigens and allergens (5). For example, in novice beekeepers, prolonged exposure to bee venom induces isotype/subclass switching to IgG4 which dampens allergic responses, rendering beekeepers resistant to bee stings (43). Moreover, chronic inflammation due to worm infections is halted by class switching to IgG4 (44). Why certain (auto)immune responses undergo a class switch to predominant IgG4 responses is not known. In MuSK MG, autoantibodies of the IgG1, -2 or -3 subclasses are monospecific and may activate complement. However, given their significantly lower levels in serum and potential agonistic effects, their contribution to the clinical manifestation in patients is uncertain (8). It is possible that in IgG4-mediated autoimmune diseases a mild IgG1-IgG3 immune response against the antigen is ongoing, similar to beekeepers. This prolonged exposure at some point may induce an autoantibody class switch to high titers of functionally monovalent IgG4 and only then do symptoms manifest. In the development of MuSK MG, high-affinity binding of MuSK antibodies may furthermore be extra critical, as functional monovalent (germlined) monoclonal MuSK antibodies lost significant binding capacity and in vitro pathogenicity (23). Affinity maturation thus seems an additional requirement for potentiating pathogenicity of monovalent MuSK antibodies. It will be exciting to learn what governs the development of IgG4 (auto)immunity.

Monovalent anti-MuSK IgG4s inhibited agrin-Lrp4-MuSK signaling in vitro and induced progressive myasthenic weakness and NMJ morphological abnormalities in NOD/SCID mice similar to polyclonal patient-purified IgG4 and monovalent Fab fragments (10, 12, 13, 21, 23, 45). This suggests that Fab-arm exchanged anti-MuSK IgG4 in polyclonal patient IgG is the main pathogenic factor causing muscle weakness in patients and animal models. Importantly, this study uses patient-derived monoclonal monovalent antibodies to model MuSK MG in vivo. These models are exciting new tools for performing preclinical therapeutic tests.

Patient-derived bivalent MuSK antibodies can potentially be pathogenic in vivo, but this differed considerably between the two antibodies studied here. The bivalent parent antibody 13–3B5 was able to cause overt myasthenic symptoms, but this required more time to start (2 to 3 wk) and progressed more slowly, as compared with monovalent 13–3B5xb12. This time course of myasthenic symptom development and progression resembles what has been reported in MuSK active immunization models (19, 35, 36, 46, 47). The MuSK antibodies induced during active immunization are functionally bivalent and monospecific, as rodent IgG is unable to exchange Fab-arms under physiological conditions (5). Furthermore, MuSK immunization of mice induces a dominant mouse IgG1 response (46). Mouse IgG1 activates complement weakly (48), suggesting that the effects of direct binding are the main cause of myasthenic symptoms upon active immunization. Therefore, the mechanism of MuSK MG in active immunization models is expected to resemble that of pathogenic monospecific, functionally bivalent MuSK antibodies like 13–3B5. Taken together, the opposing effects on MuSK signaling in vitro and the differences in time line and development of muscle weakness and the safety factor of neurotransmission in diaphragm NMJs in vivo, it would be interesting to investigate whether bivalent 13–3B5 is pathogenic through a different mechanism compared with the monovalent anti-MuSK IgG4s.

The bivalent 11–3F6 parent antibody did not induce any signs of clinical myasthenic muscle weakness, not even when doubling the exposure time and increasing the dose. Subtly different epitopes may differentially affect the conformation and activation of MuSK and, thereby, the subsequent downstream signaling and pathogenicity of bivalent MuSK antibodies. Whether nonpathogenic MuSK antibodies such as 11–3F6 or #13 (49) can mitigate the detrimental effects of pathogenic mono- and bivalent MuSK antibodies is not known.

It is important to note that a polyclonal pool of MuSK antibodies exists in patients. The question arises as to how representative the investigated clones are for the polyclonal response in different MuSK MG patients, which warrants further research. However, because the clones bind the main immunogenic region of MuSK at two different epitopes within this region (8, 15, 16), they are likely representative for a substantial part of the polyclonal response range. Some autoantibodies may be functionally bivalent, for example, when MuSK antibodies are of the IgG1, IgG2, or IgG3 subclasses or nonexchanged IgG4. These antibodies may be nonpathogenic (like 11–3F6) and may compete for binding with inhibitory monovalent anti-MuSK IgG4. Alternatively, some of these may be pathogenic (like 13–3B5) and, together with monovalent anti-MuSK IgG4, induce disease. The net result on NMJ function and disease severity will depend on the complex combination of these antagonistic and agonistic effects. It is further important to realize that the (lack of) pathogenicity of the bivalent human MuSK antibodies in this study can be mediated only by a direct effect on MuSK, as the model system used does not allow for assessment of, for example, complement activation (50). This means that it is still possible that if the subclass of the bivalent antibody allows for complement activation, it might be pathogenic in other model systems or in humans.

A critical step in this study was the generation of stable bispecific (monovalent) IgG4 antibodies. The cFAE method presented here broadens the toolbox available for generating therapeutic bispecific antibodies. By altering three residues in the Fc tail of IgG4 and adding excess of an irrelevant donor antibody (b12), <1% monospecific antibody contamination could be achieved. Furthermore, these bispecific IgG4s were stable after intraperitoneal injection in NOD/SCID mice. The field of bispecific antibody therapeutics has taken an exponential flight (51). The therapeutic promise of bispecific antibodies lies in their ability to bring together two antigens that could not be brought in close proximity with monospecific bivalent antibodies. Because of their naturally flexible structure and antiinflammatory nature, stable bispecific IgG4 have shown promise in preclinical tests for T cell redirection in the treatment of cancer and hemophilia A (52–54). Our method adds to other currently available (IgG4) antibody technology platforms and can be used for development of antibody therapies in the future.

Taken together, IgG4 Fab-arm exchange is not an innocent bystander activity, but instead potentiates the pathogenicity of MuSK autoantibodies. This may be relevant for the growing number of IgG4-mediated autoimmune diseases and other disease settings where IgG4 plays a pathogenic role, such as where IgG4 blocks endogenous antitumor responses in melanoma patients (2, 4, 55, 56).

## Materials and Methods

### Generation of Bivalent and Monovalent Recombinant Antibodies.

Anti-MuSK clones 11–3F6 and 13–3B5 were previously isolated from a MuSK MG patient and produced with a human IgG4 Fc (21). The S228P amino acid change in the anti-MuSK antibodies was achieved by converting AGC > CCC through side-directed mutagenesis based on the QuikChange II system (Agilent). To make the b12 antibody suitable as an exchange partner for cFAE, the sequence was modified to achieve S228P, F405L, and R409K amino acid changes in a pcDNA3.1 IgG4 backbone. Heavy and light chain sequences of the b12 antibody were ordered at GeneArt (Thermo Fisher). All sequences were verified using Sanger sequencing.

Recombinant monoclonal antibodies were produced in suspension FreeStyle HEK293-F cells as described previously (21) or using transient Chinese hamster ovary (CHO) cell-based expression (Evitria) (*SI Appendix*, *SI Materials and Methods*). To generate monovalent MuSK antibodies, each of the anti-MuSK clones was combined with 1.3 to 1.4x molar excess of the b12 antibody and 75 mM 2-mercaptoethylamine-HCl (Sigma-Aldrich) for 5 h at 31 °C (24). Preparations were dialyzed back to phosphate-buffered saline (PBS) using dialyzer cassettes, filter sterilized, and stored at 4 °C until use. Exchange efficiency and monovalent antibody purity were assessed using CE-MS (*SI Appendix*, *SI Materials and Methods*).

Concentrations of all recombinant antibodies were determined with nanodrop (ND-1000, v.3.8.1) using specific extinction coefficients predicted with the ProtPI tool based on the amino acid sequences (*SI Appendix*, Table S1). Furthermore, antigen-binding capacity was assessed using MuSK or gp120 enzyme-linked immunosorbent assay (ELISA) (*SI Appendix*, *SI Materials and Methods*). Antibody integrity was confirmed using PageBlue Coomassie stain according to the manufacturer’s instructions (Thermo Fisher).

### Mouse Passive Transfer Studies.

NOD/SCID mice were used to avoid a mouse immune response to the injected human recombinant IgG4. Mice were bred in the Leiden University Medical Center (LUMC) or purchased from Charles River Laboratories. They were housed in sterile, individually ventilated cages and provided with sterile food and drinking water ad libitum. Female mice were aged 8 to 10 wk at the start of the experiment unless otherwise specified.

The experimenters were blinded for the injected antibodies throughout all experiments and analyses (*SI Appendix*, *SI Materials and Methods*). To compare pathogenicity of monovalent and bivalent MuSK antibodies, mice were i.p. injected with 2.5 mg/kg recombinant antibody every 3 d. Mice were allocated to a treatment group by a laboratory member not involved in the experiment. Serum samples were taken on day 6 and 2 d after every injection. Body weight, in vivo muscle strength, and endurance were assessed daily. On day 11, mice were subjected to the end-point analyses described below.

To further interrogate possible pathogenic effects of bivalent MuSK antibodies at higher doses over a longer time, mice were injected with 5 or 10 mg/kg on days 0, 3, 7, 11, 15, 19, and 23. Serum samples were taken prior to the first injection and 2 d after every injection. Mice that received 13–3B5 were killed between day 18 and day 21 as they lost weight. Mice that received 11–3F6 were killed sequentially between day 21 and 26 due to time restrictions of the end-point analyses. For one mouse that received 11–3F6, the first injection did not lead to systemic exposure as it could not be detected in the serum. Therefore, day 0 was moved to the day of the second injection for this animal. Mice were subjected to repetitive nerve stimulation EMG on the end-point day. For the mice without signs of muscle weakness on the in vivo outcome measures, the diaphragm was prepared for measurement of ex vivo contraction force, described below. Untreated nonlittermate NOD/SCID mice (males and females aged 2 to 4 mo) were combined with the two animals that received the b12 control used as a healthy reference for EMG and contraction measurements.

Over all experiments, one mouse injected with 2.5 mg/kg 13–3B5 had to be excluded for all parameters because the serum titers revealed lower antibody levels on two time points in the experiment, likely due to misplacement of i.p. injections. For a further three mice (2.5 mg/kg b12, 2.5 mg/kg 13–3B5xb12, and 10 mg/kg 13–3B5), the measurements on the inverted mesh had to be excluded because the animals did not complete 180 s hanging during the training period.

### End-point Analyses.

After the daily measurements on the end-point day, mice were subjected to repetitive nerve stimulation EMG of the calf muscles under anesthesia, described in detail previously (10). One of the mice treated with 10 mg/kg bivalent 13–3B5 died from the anesthesia before EMG could be conducted. Substantial CMAP decrement can be measured at 40 Hz stimulation in passive-transfer MuSK MG models (10). Upon completion of the EMG, blood was collected by cutting the tail without recovery from anesthesia. Immediately thereafter, mice were killed by CO_2_ inhalation. During dissection more blood was collected via vena cava puncture. The serum from the tail and vena cava was pooled and stored at –20 °C until further analysis. The right hemidiaphragm and ETA were dissected for NMJ morphological analyses described below.

To measure ex vivo contraction force, the left phrenic-nerve hemidiaphragm was prepared as described previously (32). Briefly, the hemidiaphragm was equilibrated in Ringer’s medium. The phrenic nerve was supramaximally stimulated at 40 Hz for 7 s every 5 min until the preparation gave stable contraction. The safety factor of neuromuscular transmission was assessed by incubating the preparation with 125 nM dTC (Sigma-Aldrich) and stimulating at 40 Hz for 7 s every 5 min until the preparation gave stable contraction.

### Neuromuscular Junction Morphology.

The most dorsal strip of the right hemidiaphragm and the whole ETA were pinned up in Sylgard lined dishes and fixed in 1% paraformaldehyde (PFA) in PBS for 30 min. All incubations were done at room temperature unless otherwise specified. To enable parallel processing of all muscles in an experiment, they were stored floating in 1% PFA at 4 °C for 3 to 7 d. Before staining, muscles were blinded to the phenotypes observed in the experiment until all morphology analyses were completed. The tissues were extensively washed with PBS, remaining 1% PFA was neutralized with 0.1 M glycine in PBS (1 h), and muscles were blocked with 2% bovine serum albumin (BSA, Sigma) and 1% Triton-X (Sigma) in PBS (2 h). The muscles were subsequently incubated with 0.2 µg/mL mouse anti-SV2 (5ea, Developmental Studies Hybridoma Bank) in block overnight at 4 °C. After 6x 10 min washes with PBS, tissue was incubated with 2 µg/mL BTX-488 to visualize AChRs and 2 µg/mL Alexa Fluor 594-conjugated donkey anti-mouse IgG (A21203, Thermo Fisher) in 2% BSA in PBS for 2 h. After 6x 10 min washes in PBS, muscles were mounted in Prolong Gold mounting medium (Thermo Fisher) and stored at 4 °C until imaging. Due to technical issues with the staining, two ETA muscles (both in the b12 group) and two diaphragm preparations (1x in b12 group and 1x in 13–3B5xb12 group) had to be excluded from further analysis. Consequently, for the ETA muscles, the b12 control group was left with only two independent data points. Therefore, statistical analysis was not done on this dataset.

One high-resolution Z-stack of a representative part of the muscle was taken using a 20x objective on a SP8 confocal laser-scanning microscope with Las X software (Leica). Z-stacks were converted into maximum projections and further analyzed using ImageJ 1.52n. For the diaphragm images, 20 en face NMJs were randomly selected in the 488-BTX channel and analyzed for intensity and area using a manual threshold. For the analysis of the ETA, 30 enface NMJs were selected to better capture the variation seen in these preparations. A global threshold was manually determined for the diaphragm and ETA separately. The total AChR signal (intensity X area in the 488-BTX channel) of all analyzed NMJs per image were averaged and used as an *n* = 1 for visualization and further analysis. NMJ colocalization analysis was conducted on the ETA muscles (*SI Appendix*, *SI Materials and Methods*).

### MuSK Phosphorylation and AChR Clustering.

C2C12 myoblasts were obtained from CLS Cell Lines Service, tested for mycoplasma contamination, and maintained for a maximum of seven passages after thawing. MuSK phosphorylation and AChR clustering were assessed as described previously (21). Briefly, for MuSK phosphorylation, differentiated C2C12 myotubes were treated for 30 min. The concentration of monovalent MuSK antibodies was titered to achieve complete inhibition of agrin-induced MuSK phosphorylation. C2C12 myotubes were treated with this concentration (7.7 nM) of recombinant mono- or bivalent antibodies in the absence or presence of 0.1 nM neural agrin (R&D Systems). MuSK was immunoprecipitated from whole lysate and detected on Western blot. For AChR clustering, C2C12 myotubes were treated for 16 h with 7.7 nM recombinant antibodies in 96-well plates. After treatment, cells were stained with 2 μg/mL BTX-488 (B13422, Thermo Fisher) and 2 μg/mL Hoechst 33342 (H1399, Thermo Fisher) for 30 min at 37 °C before fixation with 4% PFA. Twenty fields divided over five wells per condition were randomly selected in the brightfield channel on a Leica AF6000 microscope. AChR cluster count and size were analyzed using ImageJ 1.52n. A manual threshold was set for each independent replicate. Large (>15 µm^2^) and all clusters >3 µm^2^ were analyzed separately. Both assays were performed in triplicate.

### Statistics.

Data are expressed as mean ± SEM. Comparisons among three or five groups were analyzed using one-way ANOVA with Šidák-corrected comparisons for parametric data. Hanging time (nonparametric data) was analyzed with the Kruskal–Wallis test with Šidák-corrected comparisons. The following comparisons were predefined: b12 vs. 13–3B5, b12 vs. 13–3B5xb12, b12 vs. 11–3F6, b12 vs. 11–3F6xb12, 13–3B5 vs. 13–3B5xb12, 11–3F6 vs. 11–3F6xb12, 13–3B5 vs. 11–3F6, and 13–3B5xb12 vs. 11–3F6xb12. Comparisons between two groups were analyzed using two-tailed unpaired *t* test. Measurements from the end-point day was used for statistical analysis by GraphPad Prism (version 8.1.1). Differences were considered significant at *P* < 0.05.

### Study Approval.

All animal studies were euthanized with approval of the Dutch national and local animal experiments committees, according to Dutch law and Leiden University guidelines.

## Supplementary Material

Supplementary File

## Data Availability

All study data are included in the article and/or supporting information.
