# Supplementary material for: Functional monovalency amplifies the pathogenicity of anti-MuSK IgG4 in myasthenia gravis
Source: Proc Natl Acad Sci U S A. 2021 Mar 22;118(13):e2020635118. doi: 10.1073/pnas.2020635118 (PMC8020787; doi:10.1073/pnas.2020635118)
Supplement: Supplementary File [file pnas.2020635118.sapp.pdf]

PNAS
www.pnas.org

Supplementary Information for

Functional monovalency amplifies the pathogenicity of anti-MuSK IgG4 in myasthenia gravis

Authors: Dana L.E. Vergoossen,[1] Jaap J. Plomp,[2] Christoph Gstöttner,[3] Yvonne E. Fillié-Grijpma,[1] Roy Augustinus,[1] Robyn Verpalen,[1] Manfred Wuhrer,[3] Paul W.H.I. Parren,[4,5] Elena Dominguez-Vega,[3] Silvère M. van der Maarel,[1] Jan J. Verschuuren,[2] Maartje G. Huijbers[1,2]*

Affiliations:

[1] Department of Human Genetics, Leiden University Medical Center, Leiden, the Netherlands.

[2] Department of Neurology, Leiden University Medical Center, Leiden, the Netherlands.

[3] Center of Proteomics and Metabolomics, Leiden University Medical Center, Leiden, the Netherlands.

[4] Department of Immunology, Leiden University Medical Center, Leiden, the Netherlands.

[5] Lava Therapeutics, Utrecht, The Netherlands.

*Corresponding authors: Dr. Maartje G. Huijbers, Einthovenweg 20, 2300 RC Leiden, The Netherlands, +31715269484 M.G.M.Huijbers@lumc.nl

This PDF file includes:

Supplementary text
Figures S1 to S7
Table S1
SI References

SI Materials and Methods

IgG purification

IgG was purified with a HiTrap MabSelect SuRe protein A affinity column (GE Healthcare) on an AKTA Pure (GE Healthcare). Antibodies were dialyzed to PBS in dialysis cassettes (Thermo Fisher) or desalted to PBS using an HiPrep 26/10 column (GE Healthcare) on an AKTA Pure, filter sterilized and stored at -20°C until use. Recombinant antibodies 13-3B5 and b12 used in all experiments were produced in HEK cells. For 11-3F6, a batch produced in HEK cells was used in Fig. 1 and 5 and Fig. S1, 2 and 5. A batch of 11-3F6 produced in CHO cells was used in Fig. 1, 2, 3 and 4, and Fig. S4, 5, 6 and 7.

Capillary electrophoresis mass spectrometry

Sheathless CE-MS was employed to assess exchange efficiency and purity of monovalent antibodies. Analyses were carried out on a CESI 8000 instrument (Sciex) coupled to an Impact Qtof mass spectrometer (Bruker Daltonics) equipped with a nanoelectrospray source. Porous-tip capillaries (91 cm x 30 µm ID) were obtained from Sciex. Capillaries were coated using polyethylenimine (Gelest) following the protocol described by Sciex (1). The background electrolyte (BGE) consisted of 10% acetic acid or 30% acetic acid and 10% MeOH for 13-3B5(xb12) and 11-3F6(xb12), respectively. Before each run, the capillary was flushed for 4 min at 100 psi with the BGE. Separation was performed by applying -20 kV at 20°C. Samples were buffer exchanged to the relevant BGE using 30 kDa MWCO filters (Vivaspin, 3 cycles of 10000xg at 4 °C) and hydrodynamically injected for 15 s using 2.5 psi. The mass spectrometer was operated in positive ionization mode using a capillary voltage of 1200 V, a drying gas temperature of 120°C and a drying gas flow rate of 1.2 L/min. An ISCID energy of 100 eV was employed to obtain proper declustering of the antibodies. Quadrupole ion and collision cell energy were 5.0 and 20.0 eV, respectively. MS control and data acquisition and analysis were performed using QTOF control and data analysis software (Bruker Daltonics). Molecular mass determinations were performed using the

Maximum Entropy deconvolution algorithm of the data analysis software. A baseline subtraction of 0.7 points was applied to the deconvoluted mass spectra.

Relative amounts of the antibodies were determined by integrating the area under the peaks observed in the BPE. For 13-3B5, the areas were directly employed to determine the relative amounts of the antibodies. For 11-3F6, different ionization efficiency was observed compared to b12 as consequence of the Fab glycans. Therefore, the relative amounts were determined by adding known amounts of the antibodies and interpolating in the corresponding calibration lines.

MuSK and gp120 ELISA

MuSK or gp120 ELISA was used to assess antigen reactivity or quantify the serum titers of the recombinant antibodies in the NOD.CB17-Prkdcscid/J (NOD/SCID) mice. To measure mono- or bivalent variants of 11-3F6 and 13-3B5, 3 µg/mL of the complete extracellular region of MuSK, produced as described previously (2), was coated on MaxiSorp plates (Thermo Fisher). For the b12 antibody, MaxiSorp plates were coated with 1 µg/mL HIV-gp120 protein (Sigma-Aldrich). Samples were diluted in an eight-point two-fold dilution series. Serum samples started at 125x dilution in block. The original batch of antibody that was injected served as a standard in duplicate; the first dilution started at 0.5-1 nM. Mouse anti-human IgG4 (Nordic clone N315, Nordic MUbio) and rabbit anti-mouse-AP (D0314, Dako) were used as secondary antibodies and conjugate respectively. Plates were developed with pNPP (VWR) and the reaction was stopped using sodium hydroxide. All samples were tested in duplicate and quantified in SoftMax pro (version 7.0.3, Molecular Devices).

Immunostaining of mouse NMJs using monovalent antibodies

To determine binding capacity of recombinant mono- and bivalent MuSK antibodies to mouse MuSK, levator auris longus muscles of NOD/SCID mice were immunostained with 2 µg/mL recombinant antibody overnight at 4°C. The preparations were co-stained for synaptic regions with 2 µg/mL

AlexaFluor594-conjugated α-bungarotoxin (BTX) (B13423, Thermo Fisher) and imaged as described previously (3).

Blinding of in vivo experiments

A researcher not involved in executing or analyzing the experiment made an inventory of body weight and assigned each mouse to a treatment group ensuring a roughly equal distribution of body weight. This researcher prepared the injection solution at the right concentration for each mouse solely labeled with the mouse code and provided this to the executing researcher. Deblinding was done after all data was collected.

Half-life and in vivo dose-finding

To determine antibody half-life, mice received a single 5 mg/kg intraperitoneal (i.p.) injection of one of the recombinant antibodies. Antibody titer was monitored in the serum obtained from blood drawn via tail vein cut. Because of animal ethical regulations, daily blood withdrawal was not allowed. Therefore, blood was drawn from mice per treatment group in an alternating fashion. Mouse 1 had blood samples (~50 µL) drawn 8, 72 and 144 hours after the injection using a tail cut. Mouse 2 had blood samples drawn 3 days before and 24, 96 and 168 hours after the injection. Samples were allowed to clot at room temperature, centrifuged at 10,000 RPM for 3 min and serum was stored at -20°C until analysis. Antibody titers were measured using the MuSK and gp120 ELISA (SI Materials and Methods). A one-phase exponential decay function with the plateau constrained at zero was fitted in GraphPad Prism (version 8.1.1) per mice to calculate the half-life. Both male and female NOD/SCID mice were used in this experiment.

To determine the minimum dose at which monovalent MuSK antibodies could be fully pathogenic, 11-3F6xb12 was i.p. injected on day 0, 3, 7, 11, 15 and 19 at doses of 1.25, 2.5 or 5 mg/kg (of the body weight at the beginning of the experiment). In addition to body weight, *in vivo* muscle strength and endurance were assessed on a daily basis using a grip strength meter and inverted mesh hang test as

described previously (4). To familiarize the mice with handling and the tests, the daily *in vivo* measurements were started 4-6 days prior to the first injection. Blood samples were taken on day -2, 5, 13 and on the final day of the experiment. Mice were killed if they lost >20% of their body weight compared to the first day of injection or when they reached the end of the experiment.

NMJ colocalization analysis

In the preparations of the ETA muscles, all *en face* NMJs were identified in the 488-BTX-channel. An NMJ was excluded if aspecific background in the SV2 channel overlapped with an NMJ. From the remaining NMJs, thirty were randomly selected for colocalization analysis with the EzColocalization plugin for ImageJ (5). Manually-determined thresholds for each channel were assigned. As a measure of innervation, the fraction of the postsynaptic AChR signal (BTX) that overlaps with the presynaptic signal (SV2), weighted for signal intensity, was quantified for each NMJ with the Mander's colocalization coefficient M1 (6). In addition, the total presynaptic signal (area x intensity in the SV2 channel) was assessed. All analyzed NMJs per image were averaged and used as an n=1 for visualization.

Protein A purification of mouse serum

To confirm the integrity of the recombinant antibodies was maintained throughout the *in vivo* experiment, they were purified back from the mouse serum with protein A. To ensure sufficient yield for CE-MS analysis, the mouse sera were pooled per condition. Protein A agarose (Roche) beads were equilibrated with PBS. Pooled serum diluted 1:1 with PBS was incubated with the protein A beads, rotating for 1 hour. Bound protein was eluted with 0.1 M Glycine HCl pH 2.5. Fractions were neutralized with 1:6 1 M Tris-HCl pH 8. Protein content was measured with nanodrop in IgG mode. Protein containing fractions were pooled and immediately processed further for CE-MS.

SI Figures

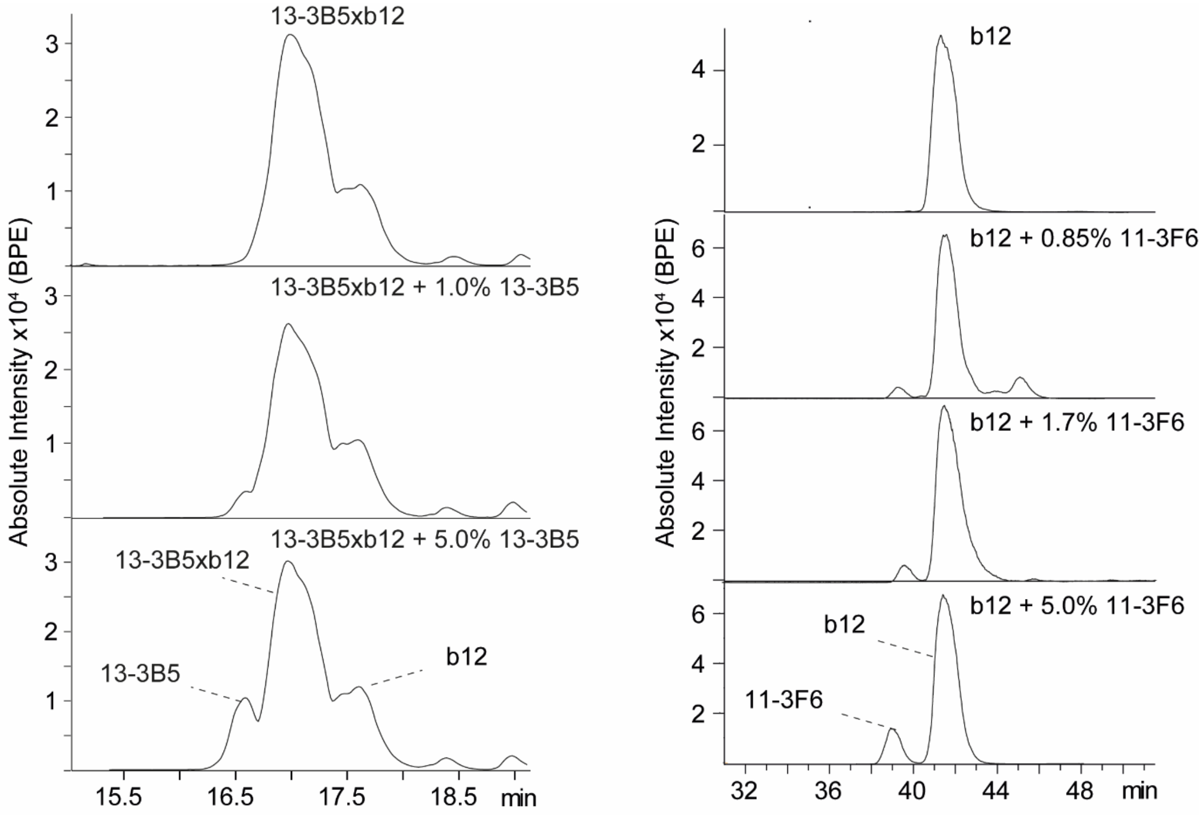

Fig. S1. Detection limit of CE-MS for bivalent 13-3B5 and 11-3F6 is below 0.5%. To assess the detection limit, antibodies were added in small amounts and analyzed by CE-MS. From the titration curve it is extrapolated that CE-MS permits determination of the bivalent antibodies in relative abundances below 0.5%.

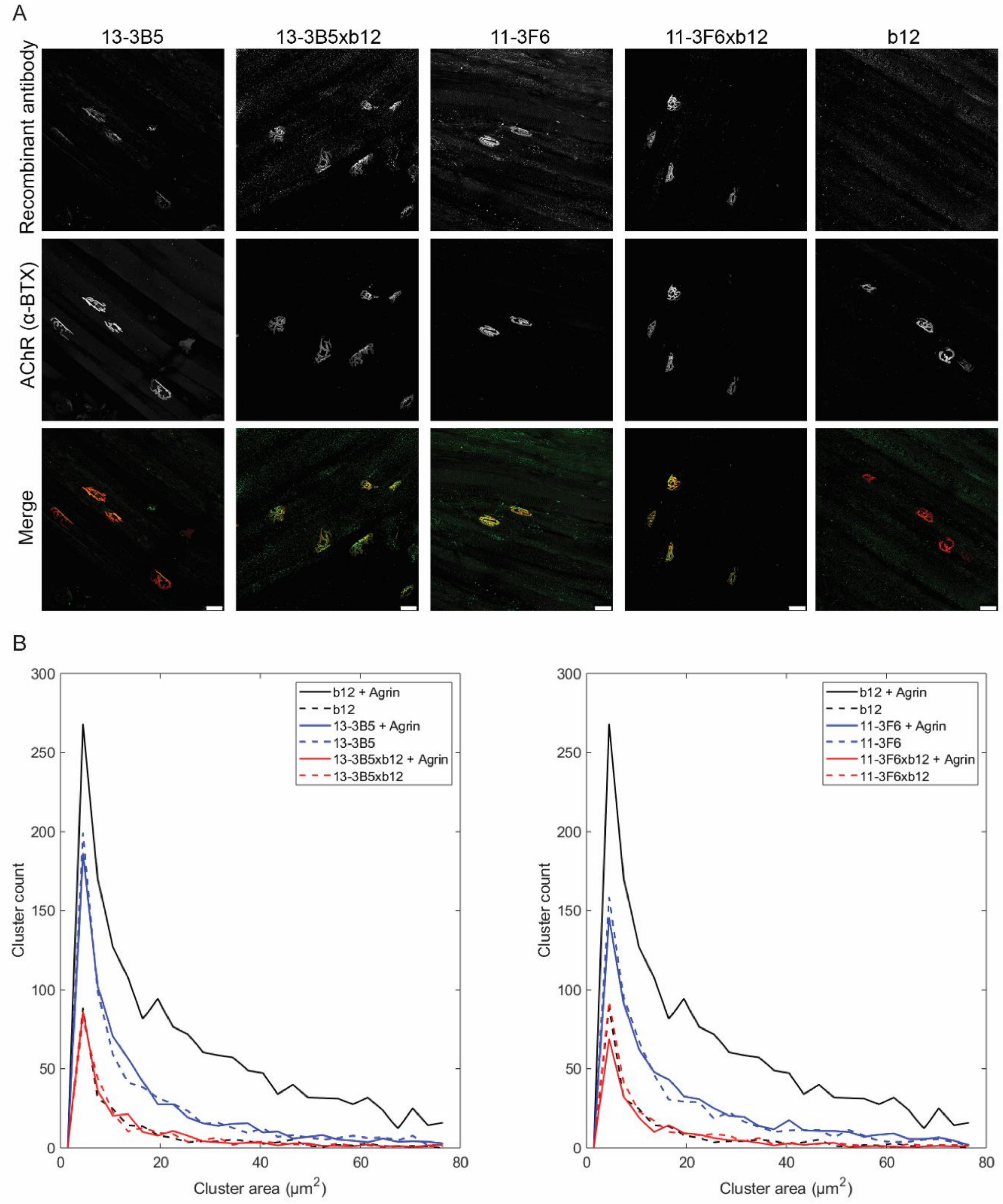

Fig. S2. ***In vitro*** **functional characterization of monovalent and bivalent IgG4 MuSK antibodies**. (A) Bivalent and monovalent MuSK antibodies bind NMJs of NOD/SCID mice. The control b12 antibody did not show binding. α-BTX = alpha-bungarotoxin. Scale bar 25 µm. (B) Distribution of AChR clusters based on cluster size (cutoff 3 μm^2) revealed that bivalent 13-3B5 induced more small clusters compared to bivalent 11-3F6 in C2C12 differentiated myotubes. The effect of the bivalent and monovalent anti-MuSK clones was independent of agrin. Distributions represent mean of three independent experiments.

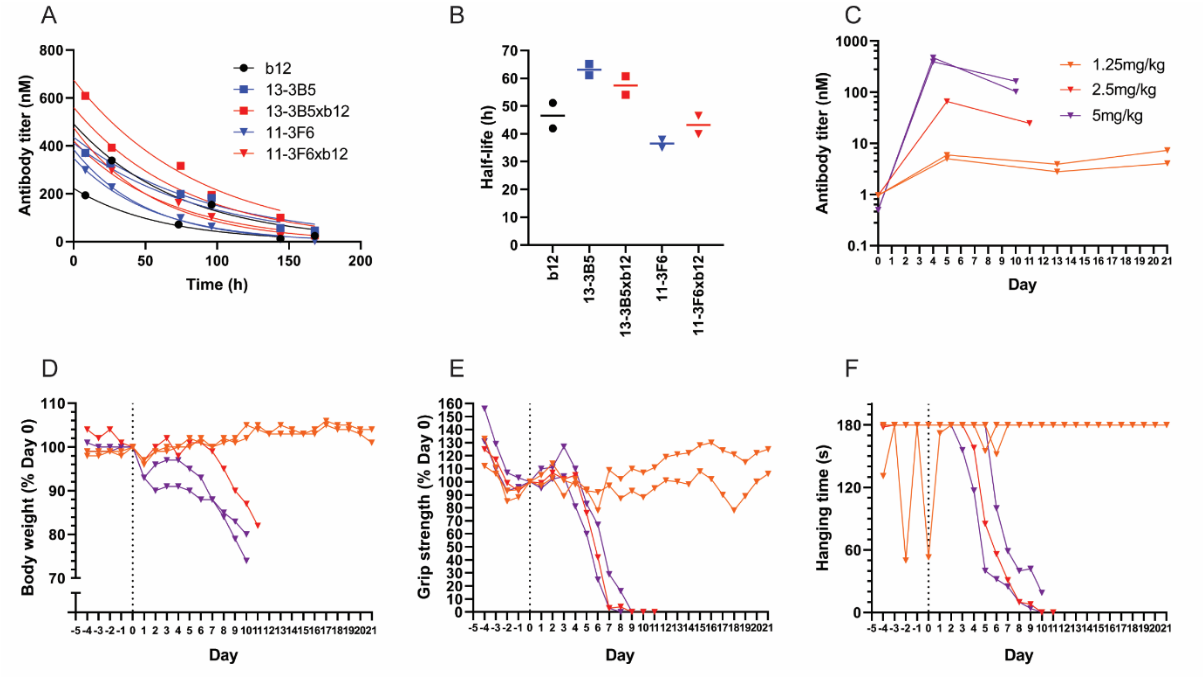

Fig. S3. Half-life and *in vivo* dose-finding. (A and B) Serum antibody titer was assessed at different times after a single i.p. dose of 5 mg/kg recombinant antibody with antigen-specific ELISA (n=2). The half-life was calculated by fitting a one-phase exponential decay function with the plateau constrained at zero. Half-life ranged between 38-63 hours, depending on the antibody. (C) MuSK antibody titers of NOD/SCID mice injected with different doses of 11-3F6xb12 every 3-4 days. (D) Body weight, (E) grip strength and (F) inverted mesh hanging time revealed that 2.5 mg/kg every 3-4 days was the minimal dose required to cause progressive phenotypical myasthenia (n=1-2). Individual data are presented.

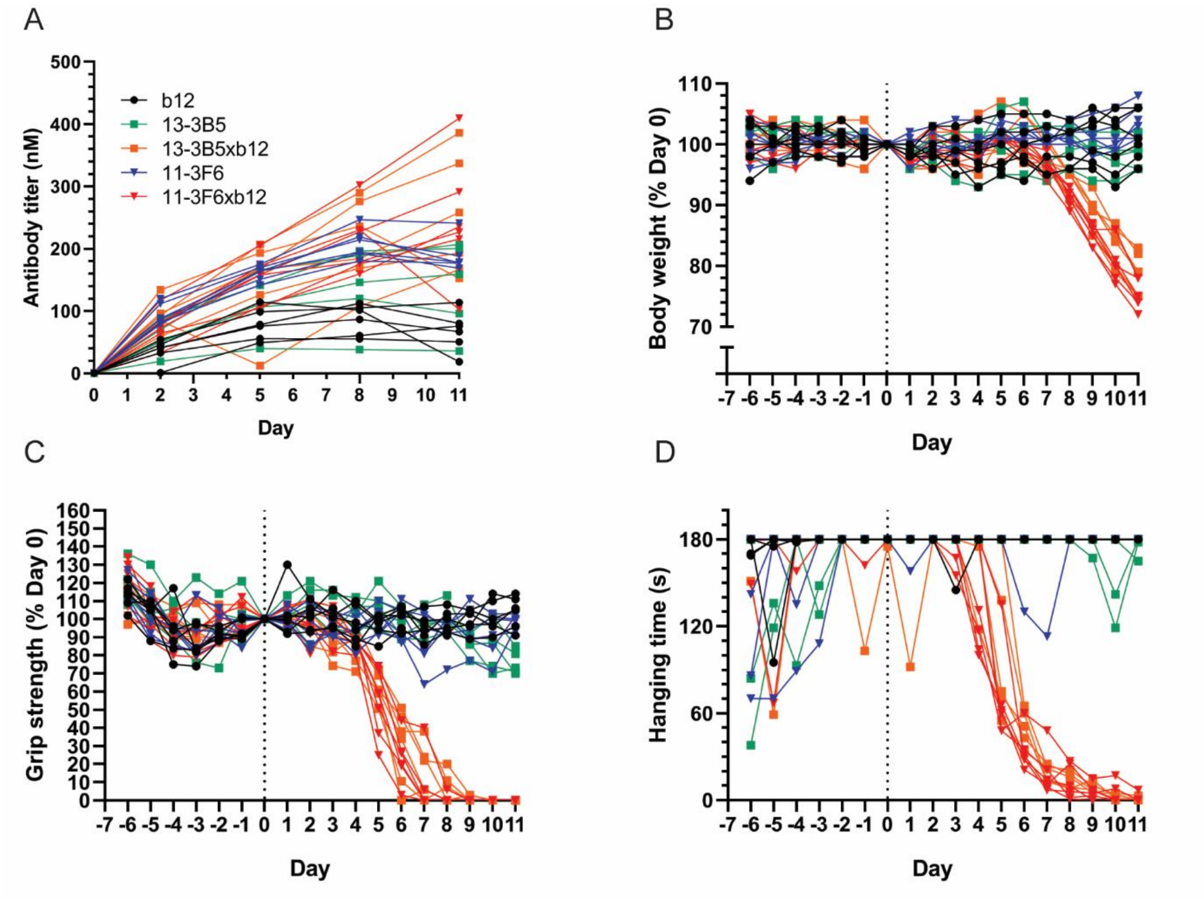

Fig. S4. Individual mouse data of *in vivo* parameters. Data trajectories of Figure 3 visualized per mouse for (A) antibody titer, (B) body weight, (C) grip strength and (D) inverted mesh hanging time. 11-3F6 and 11-3F6xb12 n=6, 13-3B5 n=5, 13-3B5xb12 and b12 n=6 (hanging time n=5).

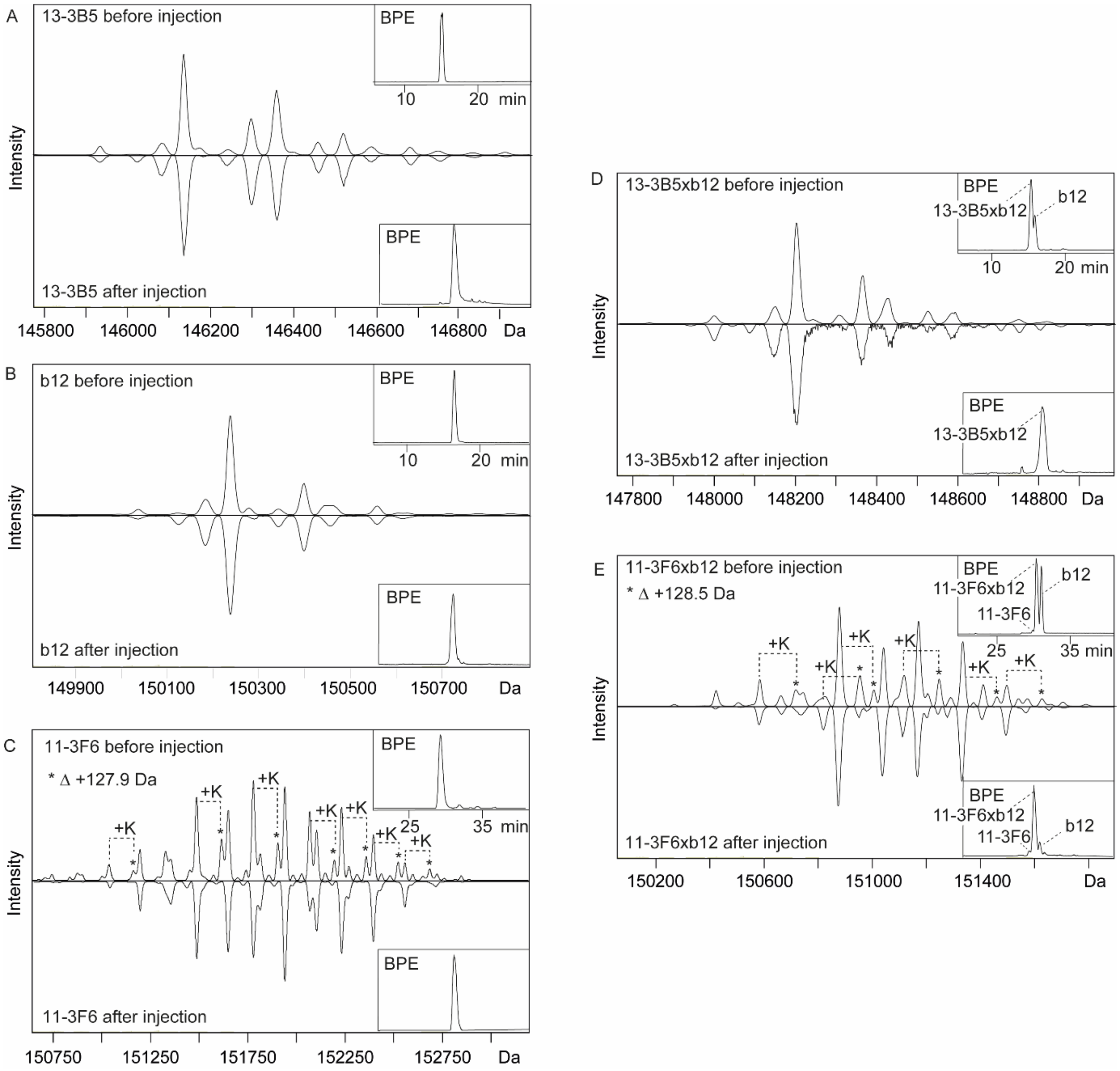

Fig. S5. Recombinant bivalent and monovalent antibodies are stable *in vivo*. Deconvoluted mass spectra and base electropherograms (BPE) of bivalent and monovalent antibodies before (upper trace) and after (lower trace) injection in mice showed stability of the antibodies *in vivo*. (A) Bivalent 13-3B5, (B) bivalent b12, (C) bivalent 11-3F6, (D) monovalent 13-3B5xb12 and (E) monovalent 11-3F6xb12. 11-3F6 (C, upper trace) and 11-3F6xb12 (E, upper trace) contained variants with an additional lysine (K) at the C-terminus of the Fc tail, indicated by (*). C-terminal lysines on IgG Fc are known to be clipped in the circulation (7) and could consequently not be observed in the mass spectra of 11-3F6 (C, lower trace) and 11-3F6xb12 (E, lower trace) purified from mouse serum.

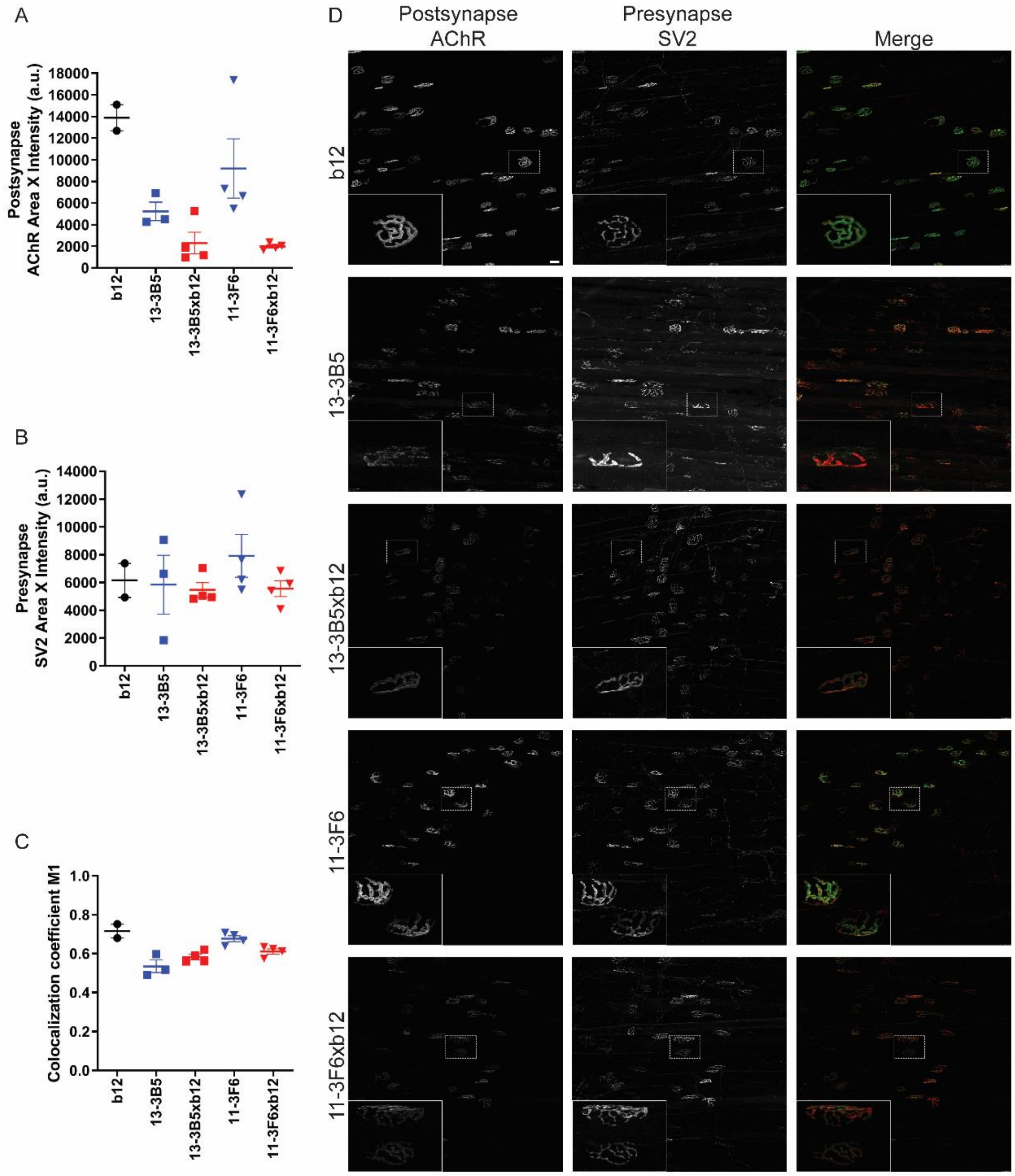

Fig. S6. Altered NMJ morphology caused by bivalent and monovalent MuSK antibodies seems to be limited to the postsynapse. AChR staining (AF488-BTX) marks the postsynaptic NMJ area and synaptic vesicle protein 2 (SV2) staining marks the presynaptic NMJ area. Thirty randomly selected NMJs per epitrochleoanconeus (ETA) muscle were analyzed and averaged. (A) Exposure to bivalent or monovalent MuSK antibodies resulted in, on average, less postsynaptic signal. (B) Presynaptic morphology does not seem to be affected by exposure to monovalent or bivalent MuSK antibodies. (C) NMJ innervation by the motor neuron was assessed by the colocalization coefficient M1, which is an intensity weighted co-occurrence coefficient of presynaptic signal (SV2) with postsynaptic signal (AChR) compared to the total postsynaptic area. Monovalent MuSK antibodies and bivalent 13-3B5 seem to slightly reduce the overlap between the pre- and postsynapse. (D) Representative maximum projections with insets per condition. In the merged picture green = AChR, red = SV2. Scalebar = 25 µm. 11-3F6, 11-3F6xb12 and 13-3B5xb12 n=4, 13-3B5 n=3 and b12 n=2. Data represents mean ± SEM.

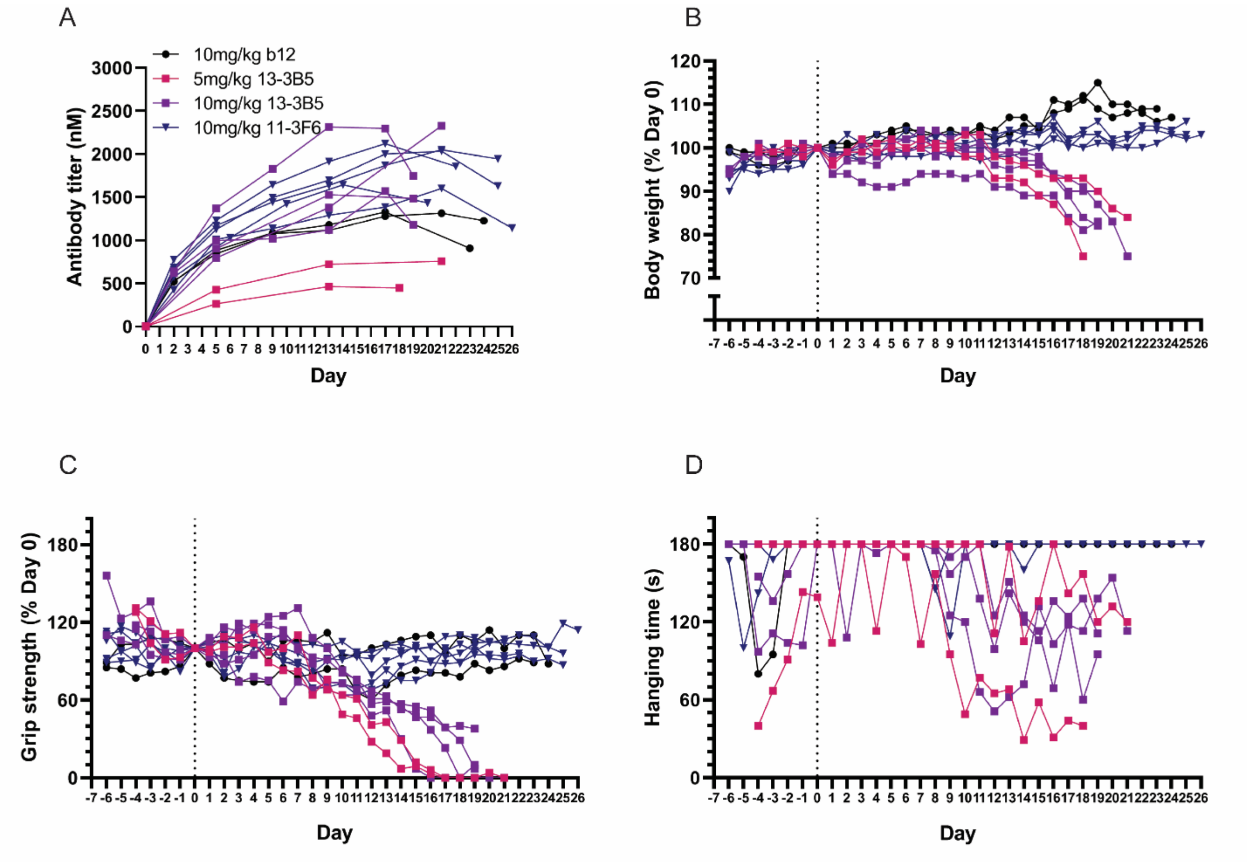

Fig. S7. Individual mouse data of *in vivo* parameters. Data trajectories of Figure 5 visualized per mouse for (A) antibody titer, (B) body weight, (C) grip strength and (D) inverted mesh hanging time. 10mg/kg b12 n=2, 5mg/kg 13-3B5 n=2, 10mg/kg 13-3B5 n=4 (hanging time n=3), 10mg/kg 11-3F6 n=5.

Table S1: Predicted molecular characteristics of recombinant antibodies, based on amino acid sequence (glycosylation was not considered).

Recombinant antibody	Mass (kDa)	Molar extinction coefficient ($x10^3$ $M^{-1}cm^{-1}$)	Absorption coefficient
**b12**	147.67	218.42	1.48
**13-3B5**	143.57	215.44	1.50
**13-3B5xb12**	145.62	216.93	1.49
**11-3F6**	144.76	196.42	1.36
**11-3F6xb12**	146.22	207.42	1.42

SI References

1. Santos MR, Ratnayake CK, Fonslow B, & Guttman A (2015) A covalent, cationic polymer coating method for the CESI-MS analysis of intact proteins and polypeptides. (Sciex - Biomarkers Omi).
2. Huijbers MG*, et al.* (2016) Longitudinal epitope mapping in MuSK myasthenia gravis: implications for disease severity. *J Neuroimmunol* 291:82-88.
3. Huijbers MG*, et al.* (2019) MuSK myasthenia gravis monoclonal antibodies: Valency dictates pathogenicity. *Neurol Neuroimmunol Neuroinflamm* 6(3):e547.
4. Klooster R*, et al.* (2012) Muscle-specific kinase myasthenia gravis IgG4 autoantibodies cause severe neuromuscular junction dysfunction in mice. *Brain* 135(Pt 4):1081-1101.
5. Stauffer W, Sheng H, & Lim HN (2018) EzColocalization: An ImageJ plugin for visualizing and measuring colocalization in cells and organisms. *Sci Rep* 8(1):15764.
6. Aaron JS, Taylor AB, & Chew T-L (2018) Image co-localization – co-occurrence versus correlation. *Journal of Cell Science* 131(3).
7. Liu H*, et al.* (2014) In vitro and in vivo modifications of recombinant and human IgG antibodies. *mAbs* 6(5):1145-1154.
